# Midgut Barrier Imparts Selective Resistance to Filarial Worm Infection in *Culex pipiens pipiens*

**DOI:** 10.1371/journal.pntd.0000875

**Published:** 2010-11-02

**Authors:** Michelle L. Michalski, Sara M. Erickson, Lyric C. Bartholomay, Bruce M. Christensen

**Affiliations:** 1 Department of Biology and Microbiology, University of Wisconsin-Oshkosh, Oshkosh, Wisconsin, United States of America; 2 Department of Pathobiological Sciences, University of Wisconsin-Madison, Madison, Wisconsin, United States of America; 3 Department of Entomology, Iowa State University, Ames, Iowa, United States of America; Center for Global Health and Diseases, Case Western Reserve University, United States of America

## Abstract

Mosquitoes in the *Culex pipiens* complex thrive in temperate and tropical regions worldwide, and serve as efficient vectors of Bancroftian lymphatic filariasis (LF) caused by *Wuchereria bancrofti* in Asia, Africa, the West Indies, South America, and Micronesia. However, members of this mosquito complex do not act as natural vectors for Brugian LF caused by *Brugia malayi*, or for the cat parasite *B. pahangi*, despite their presence in South Asia where these parasites are endemic. Previous work with the Iowa strain of *Culex pipiens pipiens* demonstrates that it is equally susceptible to *W. bancrofti* as is the natural *Cx. p. pipiens* vector in the Nile Delta, however it is refractory to infection with *Brugia* spp. Here we report that the infectivity barrier for *Brugia* spp. in *Cx. p. pipiens* is the mosquito midgut, which inflicts internal and lethal damage to ingested microfilariae. Following *per os Brugia* exposures, the prevalence of infection is significantly lower in *Cx. p. pipiens* compared to susceptible mosquito controls, and differs between parasite species with <50% and <5% of *Cx. p. pipiens* becoming infected with *B. pahangi* and *B. malayi*, respectively. When *Brugia* spp. mf were inoculated intrathoracically to bypass the midgut, larvae developed equally well as in controls, indicating that, beyond the midgut, *Cx. p. pipiens* is physiologically compatible with *Brugia* spp. Mf isolated from *Cx. p. pipiens* midguts exhibited compromised motility, and unlike mf derived from blood or isolated from the midguts of *Ae. aegypti*, failed to develop when inoculated intrathoracically into susceptible mosquitoes. Together these data strongly support the role of the midgut as the primary infection barrier for *Brugia* spp. in *Cx. p. pipiens*. Examination of parasites recovered from the *Cx. p. pipiens* midgut by vital staining, and those exsheathed with papain, suggest that the damage inflicted by the midgut is subcuticular and disrupts internal tissues. Microscopic studies of these worms reveal compromised motility and sharp bends in the body; and ultrastructurally the presence of many fluid or carbohydrate-filled vacuoles in the hypodermis, body wall, and nuclear column. Incubation of *Brugia* mf with *Cx. p. pipiens* midgut extracts produces similar internal damage phenotypes; indicating that the *Cx. p. pipiens* midgut factor(s) that damage mf *in vivo* are soluble and stable in physiological buffer, and inflict damage on mf *in vitro*.

## Author Summary

*Culex pipiens* complex mosquitoes transmit numerous diseases that affect humans and other animals. In many parts of the tropics they transmit Bancroftian lymphatic filariasis caused by the filarial nematode *Wuchereria bancrofti*. However, in parts of South Asia where Brugian lymphatic filariasis caused by *Brugia* spp. is endemic, this group of mosquitoes is present but does not play a role in transmission. The differential susceptibility of *Cx. p. pipiens* mosquitoes for *Wuchereria* but not *Brugia* species occurs as a result of the mosquito midgut environment. *W. bancrofti* larvae ingested with a bloodmeal can penetrate the *Culex* midgut, however *Brugia* larvae ingested by *Cx. p. pipiens* are unable to penetrate the midgut epithelium and die within the lumen. These observations suggest that toxic factor(s) exist within the lumen of the *Cx. p. pipiens* midgut that physically and lethally damage *Brugia* parasites. Understanding natural mechanisms of resistance to parasites in arthropod vectors is critical if we are to gain a complete understanding of the transmission dynamics and epidemiology of LF and other vector-borne diseases.

## Introduction

Lymphatic filariasis (LF) is caused by any of three mosquito-borne nematodes, *W. bancrofti*, *Brugia malayi*, or *B. timori*. Over 120 million people in 80 countries in the tropics and sub-tropics suffer are infected predominately with *W. bancrofti*, and another 1.2 billion are at risk [1]. Infection with these parasites can result in serious morbidity and can cause disfigurement of the limbs and male genitalia, i.e. elephantiasis and hydrocele [2], [3]; that leads to adverse economic and psychosexual effects. Disease elimination programs utilizing mass drug administration (MDA) in endemic areas have yielded promising results [4], but concerns exist about implementing drug administration in the absence of vector control [5], geographic expansion of the disease resulting from mass migrations from rural to urban areas [6], [7], the potential of parasite drug resistance [8], and the utility of MDA for control of zoonotic subperiodic *B. malayi*, which unlike *W. bancrofti* infects a range of non-human mammals [9], [10].

*Culex pipiens pipiens* and *Cx. p. quinquefasciatus* are principal vectors of *W. bancrofti* in urban areas of Asia, Africa, the Western Pacific, and South America [11]. These species oviposit in stagnant polluted water, and populations are increasing and expanding due to creation of favorable habitats caused by urbanization [5], irrigation [12], and in the Nile Delta, creation of the Aswan High Dam [12]. Despite their susceptibility for *W. bancrofti,* neither *Cx. p. pipiens* nor *Cx. p. quinquefasciatus* transmits *Brugia* parasites in South Asia, although natural populations are present in endemic areas [11]. Instead, nocturnally periodic *B. malayi* is primarily transmitted by *Anopheles* species, subperiodic *B. malayi* by *Mansonia* species [11], and the closely related *B. pahangi*, a coendemic filarial parasite of non-human mammals, by *Armigeres* and *Mansonia* species [13]. Several extrinsic and intrinsic factors govern the ability of a particular mosquito species to harbor and transmit a particular pathogen. Examination of vector-parasite interactions can identify potential vectors as well as provide understanding of the mechanisms underlying susceptibility and refractoriness. This information is valuable for the determination of transmission dynamics of disease in endemic areas. In this paper we define the selective barrier for *Brugia* development in *Cx. p. pipiens* that conversely has no deleterious effect on the development of *W. bancrofti* [14].

In normal development, *W. bancrofti* and *Brugia* microfilariae (mf) are ingested in a blood meal, penetrate the mosquito midgut and traverse the hemocoel to invade the thoracic muscle cells, then develop to the infective third larval stage that migrates to the mosquito head. The inability of *Cx. p. pipiens* to support the development and transmission of *Brugia malayi and B. pahangi* is apparently biological and occurs at the level of the midgut, based on observations that ingested *Brugia* microfilariae (mf) perish in the midgut soon after feeding [15], and that they can not be detected histologically or by immunohistology in extraintestinal tissues at any time point post-infection [16]. In the relatively rare case that *B. pahangi* mf do survive to penetrate the midgut and enter the thoracic musculature, development of the worms to infective third-stage larvae progresses normally; suggesting that this mosquito is otherwise physiologically compatible with *Brugia* spp. and that the midgut is the barrier to infection [17]. Here we examine the *Culex* midgut as an infection barrier, and present observations on *Brugia* mf compromised by the midgut that exhibit abnormal motility and evidence of internal damage. These studies were conducted using a laboratory strain of *Cx. p. pipiens* that was previously shown to be equally susceptible to *W. bancrofti* as the natural *Cx. p. pipiens* vector in the Nile Delta [14].

## Methods

### Parasites and parasite exposures

Sources of mf for these studies included *Brugia-*infected dark-clawed Mongolian gerbils (*Meriones unguiculatus*) infected at UW-Madison (UWM), as well as infected gerbils obtained from the NIH Filariasis Research Reagent Resource Center (FR3) at the University of Georgia, Athens; and microfilaremic blood obtained from the FR3. These *Brugia* strains, maintained for three decades by FR3, most probably originate from Koala Lampur (L. Ash and J. McCall, personal communication) and are herein referred to collectively as *Brugia*, or *Brugia* spp.). All animal use protocols were approved by UW-Oshkosh and UW-Madison Institutional Animal Care and Use committees. *Per os* exposure of *Aedes aegypti* (Black eye Liverpool strain, LVP) and an Iowa strain of *Cx. p. pipiens* to *Brugia* mf was accomplished by feeding 3- to 6-day-old mosquitoes directly on anesthetized gerbils using established procedures [18], [19]. Female mosquitoes were sucrose-starved for 8–12 hr prior to blood feeding on microfilaremic gerbils. Third-stage larvae were quantified 9–12 days post-exposure by dissecting cold-anesthetized mosquitoes in Hank's balanced salt solution (HBSS) (Fisher Scientific, Piscataway, NJ) and enumerating emerging larvae using a dissecting microscope. Infection intensity between groups was assessed using the TTEST function in Microsoft Excel (Microsoft, Redmond, WA).

### Mosquito rearing and inoculation

*Aedes aegypti* (Black eye Liverpool strain, LVP) and an Iowa strain of *Cx. p. pipiens* were maintained in a 100 sq ft walk-in environmental chamber at 26.5±0.5°C and 80±5% relative humidity. Lighting was maintained on a 16 hr light and 8 hr dark cycle with a 90 min crepuscular period at the beginning and end of each light cycle. Rearing of mosquitoes follows well-established protocols that have been detailed previously, with exposures to natural blood meals on anesthetized rabbits (LVP) and chickens (*Cx. p. pipiens*) [20], [21]. Mosquito larvae were maintained on Tetramin® fish food, fed as a slurry, and adults provided 0.3 M sucrose on cotton pads. For mosquito inoculations, *Brugia* mf were purified from fresh (<2 day old) blood samples by syringe tip filtration through 5 uM membranes (Millipore Isopore TMTP, Billerica, MA) as previously described [22]. Intrathoracic inoculation of *Brugia* mf into *Ae. aegypti* using *Aedes* saline, and *Cx. p. pipiens* using Hank's balanced salt solution (HBSS; Fisher Scientific, Pittsburgh, PA), were performed as previously described [23]. Third-stage larvae were enumerated and intensities statistically compared as described above.

### Isolation of midgut-derived mf and evaluation of parasite damage

Isolation of midgut-derived mf was accomplished by dissecting midguts from bloodfed *Ae. aegypti* within one hour of feeding because *Brugia* mf typically penetrate the midgut within 1.5 hours in this strain, and from *Cx. p. pipiens* at 2–4 hours after feeding to collect mf that displayed the compromised phenotype and were still alive. The midguts were teased apart in cold HBSS to release mf, and the mf were isolated by filtering the mixture through a syringe tip membrane as described above. Vital staining of midgut-derived mf was performed by adding an equivalent volume of 0.4% trypan blue solution (Sigma Chemical, St. Louis, MO) incubating at room temperature for 1 hr, then filtering the mf from the stain using a small syringe tip filter with a 20 µM nylon membrane (GE, Tevose, PA) into a small watch glass containing HBSS. Individual mf were transferred to slides for microscopic examination using pulled capillary needles. To enzymatically remove the sheaths from midgut-derived mf, treatment with papain was performed as previously described [24] using purified enzyme (NeuroPapain, Genlantis, San Diego, CA), and mf were recovered by filtration and wet-mounted on to glass slides in HBSS for microscopic evaluation as described above.

### *In vitro* exposure of *Brugia* mf to mosquito midgut extracts

Midguts free of foregut, hindgut, and Malpighian tubules were isolated from 3- to 6-day-old adult female *Ae. aegypti* and *Cx. p. pipiens* mosquitoes by dissection, flash frozen on dry ice, and stored at −80°C in aliquots of 50 midguts per 0.6 mL tube. Extracts were made by suspending midguts in chilled HBSS on ice at a ratio of 0.5 µL buffer to 1 midgut, then compressing the midguts with a 0.5 mL Kontes pellet pestle (Fisher Scientific, Pittsburgh, PA) using ∼10 gentle presses. The mixture was microcentrifuged at 5,000× g for 5 minutes at 4°C to pellet the midguts. The supernatant was removed to a new 1.5 mL tube and microcentrifuged at 10,000× g for 5 minutes at 4°C to pellet remaining particulates. The cleared crude extract was aliquotted into sterile 0.6 µL tubes and stored at −80°C. Blood-derived *B. malayi* mf were filtered and concentrated into a small volume of HBSS as described above, and were added to thawed midgut lysates in a sterie 0.6 µL tube at ratios of ∼6 mf per midgut. The mf were incubated at 26°C for 2 hours, then were transferred to glass slides for microscopic examination, or subjected to vital staining or papain treatment.

### Scanning electron microscopy

Midgut-derived mf were fixed on 0.2 µm syringe tip silver filters with 2.5% glutaraladehyde prepared in 0.05 M sodium phosphate buffer (SPB) overnight, washed twice with SPB, dehydrated through a graded ethanol series, and then critical point dried (Tousimis Samdri-780A, Rockville, MD). The specimens were Sputter Coated with a ∼25 nm layer of gold/palladium and imaged with an SEM accelerating voltage at 10 kv (Hitachi S-570, Pleasanton, CA).

### Transmission electron microscopy

Midgut-derived mf were fixed in Karnovsky's fixative (2.5% glutaraldehyde/2.0% formaldehyde in 0.1 M NaPO_4_ buffer (PB, pH = 7.2) at 4°C. For ease of specimen handling (and to prepare the samples for flat embedding) the following steps were followed. Samples were lightly vacuumed onto 0.4 µm filters and enrobed in molten 2% low temperature agarose cooled to ∼50°C. The agarose was lightly pressed onto the sample into sheets and immediately cooled on a pre-chilled aluminum block (−20°C). Excess bare agarose was dissected and discarded with all remaining steps performed on the specimens in glass vials on a rotator. The agarose samples were placed into fresh Karnovsky's fixative for 2 hours and post-fixed with 1% OsO_4_ in PB for 1 hour at RT. The samples were dehydrated through a graded ethanol series and embedded in Spurr's low viscosity resin (ERL 4221 formulation, Polysciences Inc. Warrington, PA). Specimens were sectioned on a Leica UC6 ultra-microtome, stained in uranyl acetate and Reynolds lead citrate and viewed on a Philips CM120 (FEI Co. Eindhoven, Netherlands) at 80 kV. Images were collected on an Olympus-SIS MegaView III (Olympus-SIS Corp., Lakewood, CO) digital camera.

### Video capture and photo editing

Mf motility was observed with an Olympus SZH10 zoom stereomicroscope, with maximum magnification of 70×. Images were visualized using the attached DC-330 color camera (Dage-MTI Inc., Michigan City, IN), with signal conversion from S-video to DV by an ADVC-55 digital video converter (Green Valley/Canopus), and MPEG4 videos were captured using MPEGCraft 3 DVD version 3.03 (Canopus). Original video clips were imported into iMovie '09 version 8.0.6 (Apple Inc.) to edit for run time and to highlight movements of particular worms by cropping them out of specified fields. Final videos were converted to ACC files. See Video S1 and S2. Digital micrographs were labeled using Adobe Photoshop CS5 (Adobe Systems Incorporated, San Jose, CA).

## Results

### Compatibility of *Cx. p. pipiens* for *Brugia* spp

Less than half (43–46%) of the *Cx. p. pipiens* exposed to *B. pahangi-*infected gerbils became infected, compared to 95% in *Ae. aegypti*. For sake of comparison, when this *Culex* strain was exposed to *W. bancrofti* collected from human volunteers in the Nile Delta, infection prevalence ranged from 59.2% and 61.2% prevalence [14]. Infection intensities in *Cx. p. pipiens* also were statistically lower than for *Ae. aegypti* as determined by testing of the null hypothesis by two-tailed Student's T-test (p<0.001) (Table 1). *Cx. p. pipiens* was, however, almost completely refractory to *B. malayi*, with a single third-stage larva found in a mosquito exposed to a very high microfilaremia. Because *B. pahangi* is more easily propagated in the laboratory, and it is easier to collect sufficient numbers of *B. pahangi* mf; we chiefly used *B. pahangi* for downstream experiments. To determine if *Cx. p. pipiens* is physiologically compatible for *Brugia* infection, we bypassed the midgut by inoculating blood-derived mf directly into the hemocoel of adult female mosquitoes. Introducing blood-derived *B. pahangi* mf directly into the *Cx. p. pipiens* hemocoel resulted in thoracic muscle invasion and normal larval development to intensities comparable to control mosquitoes (p>0.1 with Student's T-test for unpaired samples) (Table 2). We observed similar results in an unreplicated inoculation experiment with *B. malayi* (prevalence in *Cx. p. pipiens* 76% with intensity of 2.1 L3/mosquito, n = 21; *Ae. aegypti* prevalence 100%, intensity 6 L3/mosquito, n = 20). Midgut-derived *B. pahangi* mf from *Cx. p. pipiens*, however, failed to develop when inoculated into the susceptible *Ae. aegypti* strain (Table 2), indicating that damage incurred within the midgut is lethal. In all inoculation experiments, a subset of mosquitoes in each group was dissected within 3 hours of inoculation to verify that mf were successfully introduced (data not shown).

**Table 1:** Development of *Brugia* spp. parasites in mosquitoes following blood feeding on microfilaremic gerbils.

		Prevalence and mean intensity of L3s in mosquitoes at 9–12 DPI^a^	
Parasite	Microfilaremia	*Ae. aegypti* LVP	*Cx. p. pipiens* IA
*B. pahangi*	26 mf/20 µL	95% (20)^b^12.0±5.5^c^	46% (13)2.0±1.7
	45 mf/20 µL	95% (21)10.0±8.8	43% (21)7.0±7.5
*B. malayi*	61 mf/20 µL	95% (19)6.0±4.6	0% (19)0
	198 mf/20 µL	100% (22)9.0±6.6	5% (22)1.0

a DPI  =  Days post ingestion.

b Prevalence of infection indicates the percentage of bloodfed mosquitoes infected. The total number of mosquitoes dissected in each group is provided in parentheses.

c Intensity indicates the mean and standard deviation of L3s in infected mosquitoes.

**Table 2:** *Brugia pahangi* mf successfully develop to infective stage larvae when inoculated into the hemocoel of *Cx. p. pipiens*, and fail to develop after exposure to the *Culex* midgut environment.

Mf source	Exposure to mosquito midgut?	No. of mf injected	Prevalence and mean intensity of L3s in mosquitoes at 9–12 DPI^a^	
			*Ae. aegypti* LVP	*Cx. p. pipiens* IA
Blood-derived	None	30–50 mf/mosq.	100% (10)^b^12.0±5.4^c^	100% (5)12.0±4.0
		10–30 mf/mosq.	100% (20)14.0±8.2	n.d.^d^
		10–20 mf/mosq.	n.d.	95% (20)7.0±5.4
LVP-midgut	0.5 h in LVP	∼10 mf/mosquito	100% (5)5.0±2.3	n.d.
		∼10 mf/mosquito	75% (21)3.0±1.6	n.d.
Cpp-midgut	1.5 h in Cpp	∼10 mf/mosquito	0% (20)	n.d.
		∼10 mf/mosquito	0% (21)	n.d.

a DPI  =  Days post inoculation.

b Prevalence of infection indicates the percentage of bloodfed mosquitoes infected. The total number of mosquitoes dissected in each group is provided in parentheses.

c Intensity indicates the mean and standard deviation of L3s in infected mosquitoes.

d n.d.  =  not determined.

### Internal damage of *Cx. p. pipiens*-derived *B. pahangi* mf

*B. pahangi* mf recovered from *Cx. p. pipiens* midguts displayed compromised motility and in some cases kinked posture, characterized by stiffening of worms that bent at angles instead of displaying the sigmoidal thrashing movement characteristic of viable, *Ae. aegypti* (LVP)-derived mf (Supplements S-1and S-2). This effect also was observed in mf that were incubated in soluble *Cx. p. pipiens* midgut extracts *in vitro* (data not shown), and presumably arose from deleterious effects of the midgut environment on mf tissues. Internal damage was visible in midgut-derived mf observed by light microscopy in the form of visible internal vacuolization, which was further investigated by vital staining in 0.4% trypan blue. Healthy *Ae. aegypti* midgut-derived mf did not internally absorb stain, but in some cases stain was observed in the space between the sheath and the cuticle at the head or tail (data not shown). The vital stain, however, freely crossed the cuticle of *Cx. p. pipiens* midgut-derived *Brugia* mf and stained internal body cells, often within the central third of the worm length, providing evidence of internal cell death.

**Figure 1:**
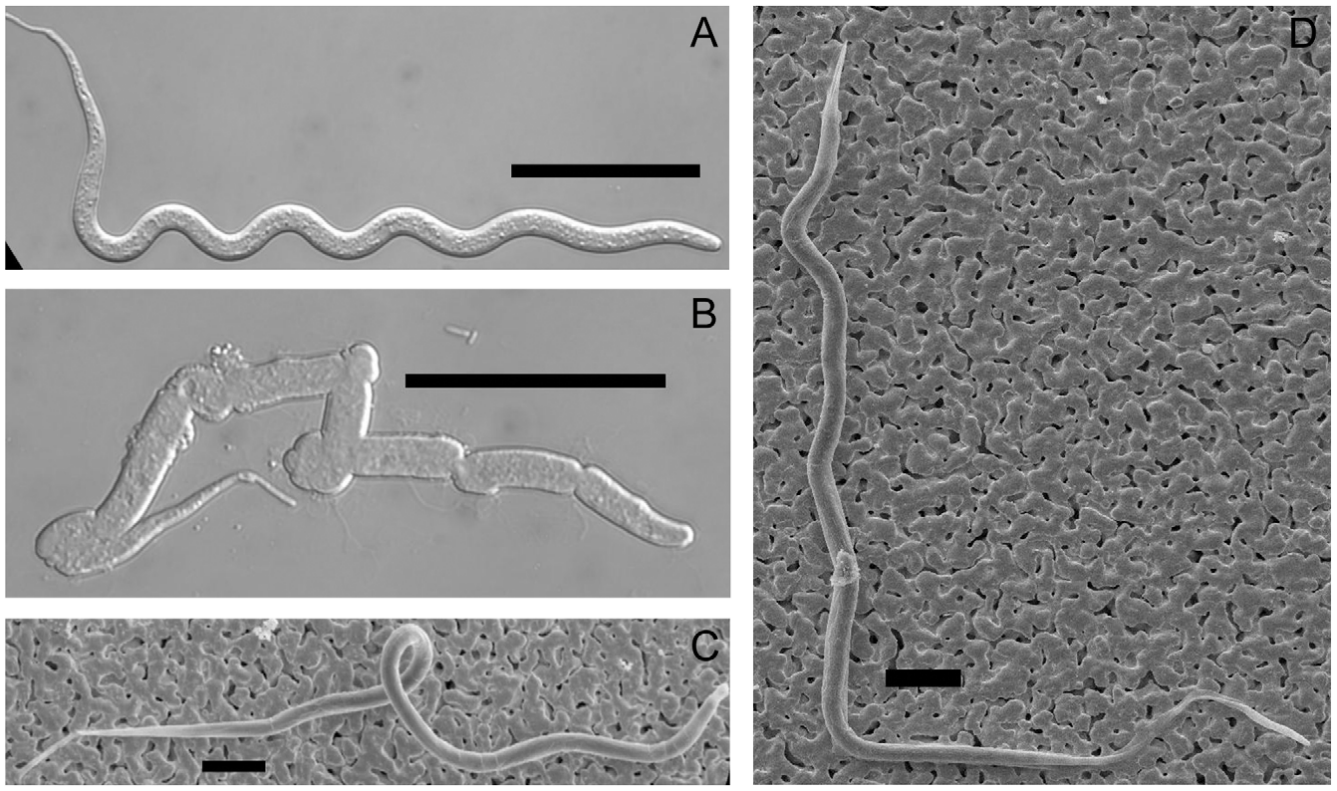
Enzyme sensitivity and external morphology of midgut-derived *B. pahangi* mf. Panel A, LVP-derived mf with sheath removed by papain treatment; B, Cpp-derived mf after papain treatment; C, scanning electron micrograph of sheathed LVP-derived mf; D, scanning electron micrograph of sheathed Cpp-derived mf. Scale bars: panels A and B, 50 µM; C and D, 20 µM.

**Figure 2:**
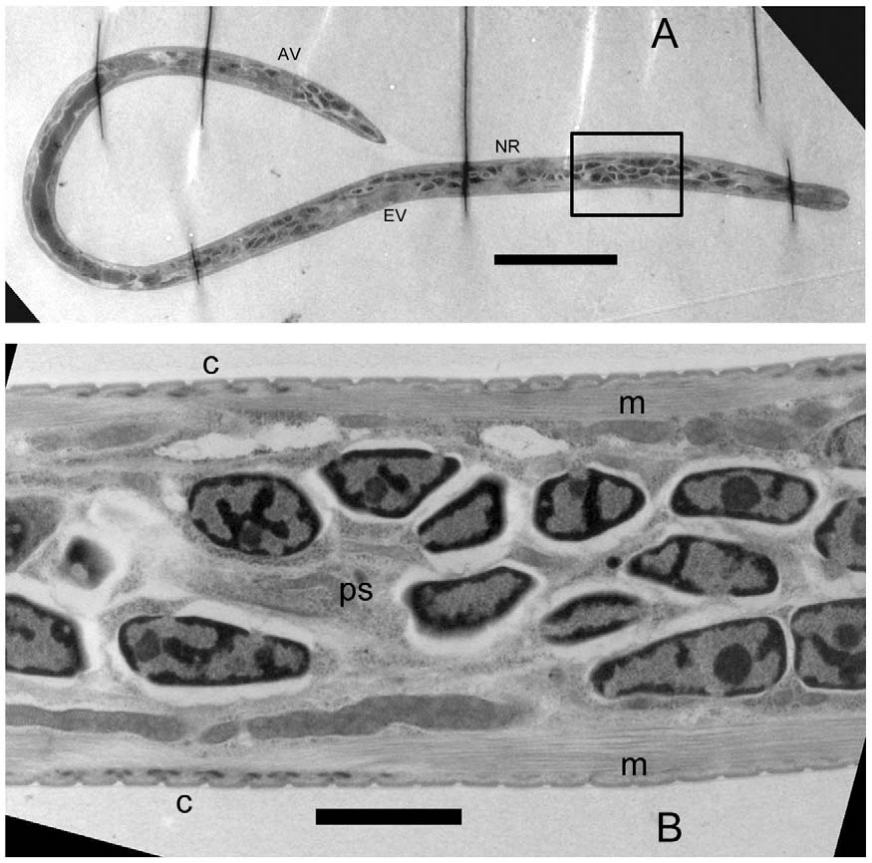
Ultrastructural aspects of LVP-derived *B. pahangi* mf. Longitudinal section demonstrates typical ultrastructural aspects of a healthy mf, with pronounced nuclei in the nuclear column, regular striations in the cuticle, and undisrupted longitudinal body muscle. Panel A, longitudinal section of full length mf; B, high magnification view of nuclear column in the boxed area anterior to the nerve ring. NR, nerve ring; EV, excretory vesicle; IN, innenkorper; AV, anal vesicle; C, scalloped cuticle; m; longitudinal muscle; ps, pseudocoelom. Scale bars: panel A, 20 µM; B, 2 µM.

**Figure 3:**
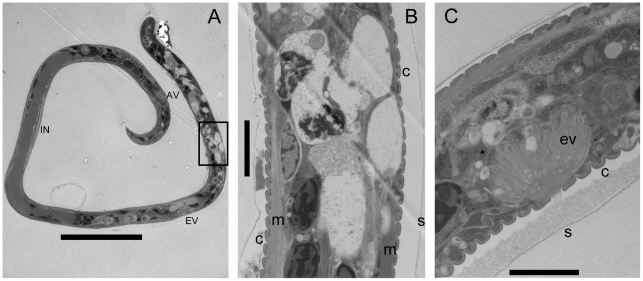
Ultrastructural aspects of Cpp-derived *B. pahangi* mf. Longitudinal section demonstrates vacuolization of the nuclear column, disruption of the hypodermis and body wall muscle, and release of material from the excretory vesicle. Panel A, longitudinal section of full length mf; B, high magnification view of nuclear column in the boxed area anterior to the excretory vesicle; C, excretory vesicle activity from a Cpp-damaged worm, showing release of visible material from the pore and accumulation of the material between the scalloped cuticle and the overlying sheath. NR, nerve ring; EV, excretory vesicle; IN, innenkorper; AV, anal vesicle; C, cuticle; m; longitudinal muscle. Scale bars: panel A, 20 µM; B and C, 2 µM.

*Brugia* mf are covered with a vestige of the eggshell membrane that forms a membranous chitinous sheath. A standard procedure to enzymatically remove the sheath is to treat mf with 10 mg/mL papain, which successfully removes the sheaths from blood-derived mf with no harm to the worms [24]. In an effort to visualize the underlying cuticle of midgut-derived *B. pahangi* mf, we subjected them to papain treatment to remove the sheaths, and found that treatment efficiently removed the sheaths of blood and *Ae. aegypti*-derived mf with no harm to the worms, but completely dissolved *Cx. p. pipiens*-derived mf. At 1/10 the recommended papain concentration, most control mf were exsheathed and all were motile, but many *Cx. p. pipiens*-derived mf were fatally damaged with bulges in the body wall, and spilling of body contents from multiple regions across the body wall (Figure 1A and B); indicating that damage inflicted by the toxic midgut environment weakened the body wall of *Cx. p. pipiens*-derived mf to enzymatic attack. Scanning electron microscopy of seven intact midgut-derived *B. pahangi* mf from each vector species further underscored the kinked phenotype in *Cx. p. pipiens*-derived worms (6 from *Cx. p. pipiens* versus 2 from *Ae. aegypti*) and bagginess of the sheath around *Cx. p. pipiens*-derived worms (5 *Cx. p. pipiens*-derived mf, 1 *Ae. aegypti*-derived) (Figs. 1C and D), and apparent cuticular constrictions in bent regions of *Cx. p. pipiens*-derived worms (4 *Cx. p. pipiens*-derived, 0 *Ae. aegypti*-derived) (data not shown), however direct visualization of the worm surfaces was not possible because of the presence of the overlying microfilarial sheath. Bagginess of the sheath was also observed in longitudinal TEM sections of *Cx. p. pipiens*-derived worms, however at this level we observed no evidence for cuticular damage or constriction. The most notable ultrastructural observations of sectioned worms was the marked accumulation of vacuoles disrupting the nuclear column and body wall of *Cx. p. pipiens*-damaged *B. pahangi* mf (Figs. 2 and 3A,B), disruption of the hypodermis underlying the cuticle (data not shown), and in one case release of visible contents from the excretory vesicle (Fig. 3C).

## Discussion

Previous work has demonstrated that *Brugia* mf fail to penetrate the midgut of *Cx. p. pipiens* complex mosquitoes and die in the midgut lumen [15], [25]; that *W. bancrofti* mf ingested by *Cx. p. pipiens* are not damaged by the cibarial armature or inhibited from midgut penetration by peritrophic membrane formation [26]; and that different mosquito species and/or strains within this mosquito complex are either completely or partially refractory to *B. pahangi* [17], [27], [28]. Here we present experimental support for these observations and for our hypothesis that the *Cx. p. pipiens* midgut acts as an innate and selective barrier to infection with *B. malayi* and *B. pahangi.* In our studies, the damage inflicted on *Brugia* mf within the *Cx. p. pipiens* midgut was largely lethal in nature and precluded further development in extra-intestinal tissues (i.e. thoracic muscles). Our experimental infection data clearly demonstrate that *Cx. p. pipiens* is otherwise physiologically compatible for *Brugia* mf, and that the midgut barrier is comparatively more restrictive for *B. malayi* than for *B. pahangi*. The mechanism of *Cx. p. pipiens*-induced midgut damage to *Brugia* mf is not yet clear but the differential vital staining and protease sensitivity of intact (*Ae. aegypti*-derived) and damaged (*Cx. p. pipiens*-derived) worms indicate that the *Cx. p. pipiens* midgut environment apparently breaches the mf cuticle, leading to death of cells inside the worms. The subcuticular damage evident in our ultrastructural studies provide insight regarding the compromised ‘kinked’ movements that are observed in *Brugia* mf exposed to the *Cx. p. pipiens* midgut.

The mosquito midgut epithelium is one of the first physical barriers encountered by ingested pathogens; it is composed of a single layer of polarized epithelial cells supported by an underlying basal lamina [29], [30]. The midgut epithelial cells form a microvillar surface on the lumenal side and secrete digestive enzymes into the lumen upon ingestion of a bloodmeal. In mosquito-arboviral systems, midgut infection and escape barriers that have been described include: inhospitable chemical environment of midgut lumen that destroys incoming virions (e.g., proteolytic enzymes, pH), lack of epithelial receptors for viral attachment and/or entry, dose dependence of epithelial cell infection, and relative abundance of organelles necessary for virion assembly [31]. These intrinsic barrier systems are geneticially controlled, and can be expressed in variable proportions wthin a mosquito population, thereby affecting epidemiology of disease. Mesenteric (midgut level) barriers also exist in mosquito species exposed to eukaryotic pathogens. For example, in the case of *Plasmodium* parasites that cause human and avian malaria, destruction of ookinetes by digestive enzymes can occur in the midgut lumen, increased nitric oxide production and superoxide anion production can kill ookinetes, ookinetes can be killed by pattern-recognition receptor mediated phagocytosis [32], the absence of molecular recognition sites on midgut cells can prevent ookinete invasion [33], intracellular *Plasmodium* ookinetes can be lysed [34], oocysts can be targeted by phagocyte attack, and oocysts can be encapsulated and melanized [32]. Successful pathogen development is clearly dependent on vector cells and molecules, and on the genetic makeup of the pathogen itself, as evidenced by the selection of arboviruses for attenuation in mosquito vectors and vertebrate hosts [31], differential susceptibility of culicine and anopheline mosquitoes to avian and human malaria parasites [34]; and the selection of filarial worms for increased infectivity in a permissive vector [35].

The *Cx. p. pipiens-Brugia* barrier described here is the second filarial midgut barrier reported from mosquitoes that naturally transmit filarial worms (the first demonstrated that substantial numbers of *Dirofilaria immitis* mf are retained in the midgut of *Aedes trivitattus* [36]; and like other described mosquito midgut barriers to viral and eukaryotic pathogen infection, it is expressed at the intra- and interspecific levels [17], [27], [37], and is undoubtedly under complex genetic control. One of the most highly characterized filarial infection barriers is the physiological incompatibility observed in the yellow fever mosquito *Ae. aegypti*, which is controlled by at least two loci [38]; the major being a sex-linked recessive gene designated *f^m^* [39]. The LVP strain of *Ae. aegypti* that is routinely used to maintain laboratory *Brugia* strains was selected for susceptibility to subperiodic *B. malayi*, and is also susceptible to *B. pahangi* and *W. bancrofti*, but not to *Dirofilaria immitis or D. repens* [40]. Filarial worm susceptibility in *Cx. pipiens* complex mosquitoes is also controlled genetically, but is different than that reported for *Ae. aegypti*. In direct contrast to the *f^m^* gene of *Ae. aegypti*, the *sb* gene of *Cx. p. pipiens* influences the susceptibility of *Cx. p. pipiens* for *Brugia* but not *W. bancrofti* [41]. The genetics of filarial susceptibility are likely more complex for *Culex pipiens* complex mosquitoes than for *Ae. aegypti* because Egyptian *Cx. p. pipiens* populations can be selected for higher susceptibility but not refractoriness for *W. bancrofti* [42], and similarly for *Cx. p. quinquefasciatus* [43]; therefore, it is not surprising to find that the mechanisms of refractoriness could significantly differ between *Aedes* and *Culex* vectors. Extensive studies on susceptibility of *Cx. tarsalis* (*Cx. sitiens* complex) for Western equine encephalitis virus also indicate complex genetics underlying vector competence of a congeneric organism with a midgut infection barrier for a viral pathogen [44].

Most LF elimination efforts follow the WHO-recommended mass administration regimen of treating individuals in endemic areas with anti-filarial drugs annually, to eliminate microfilaria production and prevent transmission to mosquito vectors [45]. In many cases these programs have drastically dropped microfilaremias, providing a cost-effective strategy to improve health on a broad scale and targeting multiple parasitic infections simultaneously [4]. In very few of these localities, however, are vector control efforts being integrated with chemotherapeutic control [5], despite the observations that implementing vector control practices with MDA can influence key ecological parameters that further sustain LF elimination [5], [46]; and that in some regions LF transmission has returned after cessation of MDA [47], [48]. Because ecological and geographical conditions vary greatly between endemic areas, the length of time that the MDA strategy must be continued to eliminate LF transmission is difficult to calculate, is likely region-specific, and depends on many factors including biology of the vector(s) involved [45], [46], particularly if xenomonitoring is being considered as a tool for program assessment. It is clear that the complex dynamics that govern parasite transmission vary significantly between endemic regions, and that efforts to achieve elimination of LF must be based on local transmission thresholds - to do this, local transmission dynamics must be understood, and this demands understanding of the biology of vector and nonvector mosquito species in these areas, and the competence of local mosquito strains to successfully transmit the parasites.

## Supporting Information

Video S1 This uncompromised movement is characteristic of *Brugia* mf recovered from the midgut of *Aedes aegypti* LVP strain.
(6.86 MB MOV)

Video S2 Compromised, or kinked, motility characteristic of *Brugia* mf isolated from the *Cx. p. pipiens* midgut.
(7.07 MB MOV)
